# Not Another Presentation of Cellulitis: A Case Report of Erythromelalgia

**DOI:** 10.21980/J8BD2K

**Published:** 2022-01-15

**Authors:** Raymen Rammy Assaf, Kelly Winters

**Affiliations:** * Harbor UCLA Medical Center, Department of Pediatric Emergency Medicine, Torrance, CA; ^ Children’s Hospital of Orange County, Emergency Medicine Specialists of Orange County, Orange, CA

## Abstract

Cellulitis is among the more common conditions diagnosed and treated within pediatric emergency departments. However, unusual distribution, recurrence, and/or poor response to antibiotics may point towards another pathologic process. This case presentation illustrates a teenager with recurrent episodes of painful erythematous and swollen extremities misdiagnosed repeatedly as cellulitis. Careful history, exam, laboratory evaluation and rheumatologist consultation aided in making the correct diagnosis of erythromelalgia, which was successfully treated with supportive measures. This report reviews a rare presentation of erythromelalgia, as well as its pathophysiology, recommended therapies, and common misdiagnoses.

**Topics:**

Painful extremities; recurrent swelling; erythromelalgia.

[Fig f1-jetem-7-1-v31] [Fig f2-jetem-7-1-v31]

**Figure f1-jetem-7-1-v31:**
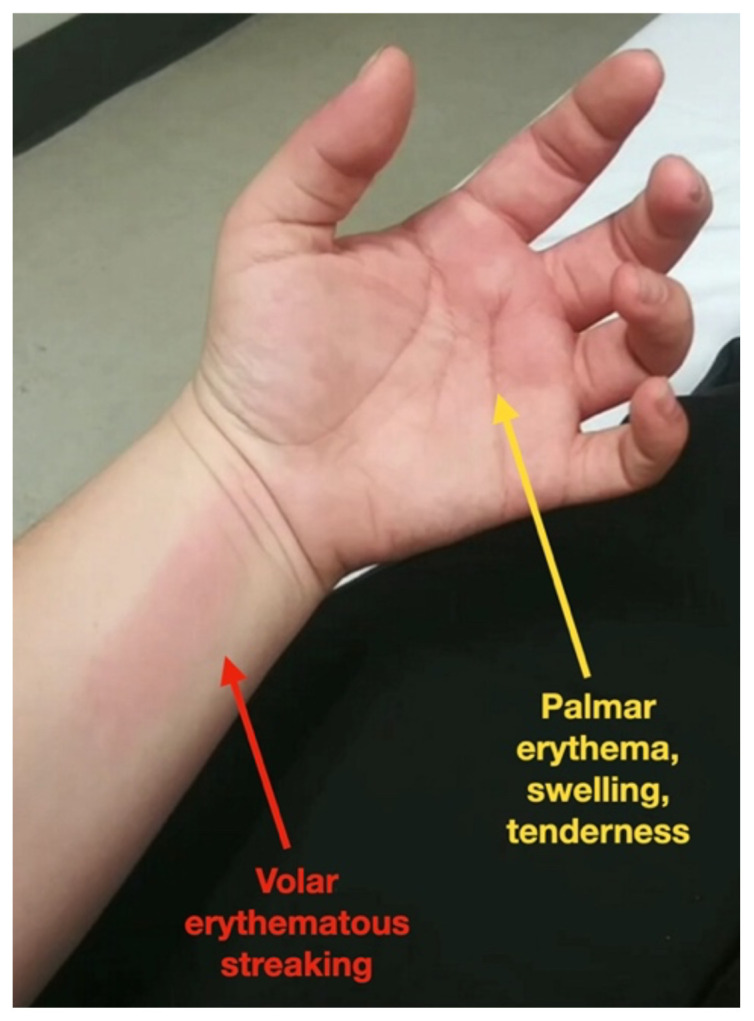


**Figure f2-jetem-7-1-v31:**
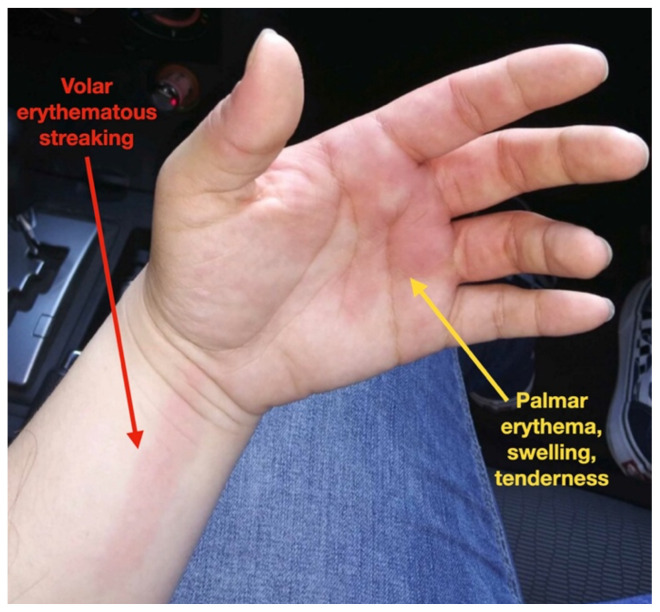


## Brief introduction

Erythromelalgia is an exceedingly rare but often debilitating disease due to neurovascular dysregulation of the distal extremities, with incidence rates of 2 per 100,000 females and 0.6 per 100,000 males.^1^–^5^ The diagnosis may be elusive in the emergency department (ED) setting due in part to its rarity and resemblance to more common diseases such as cellulitis, lymphangitis or Raynaud phenomenon.^1^,^2^ This report illustrates the case of a teenage female presenting to the ED with episodic extremity pain, swelling and erythema initially diagnosed and treated as recurrent cellulitis. The featured images of this patient’s presentation depict the more characteristic appearance of erythromelalgia, and the case report reviews literature on the pathologic features and management of this sparsely described disease.

## Presenting concerns and clinical findings

A 17-year-old woman with history of “recurrent cellulitis” presented with intermittent painful, red, warm and swollen hands and feet. She has had four previous episodes over the last five years, all of which were treated with inpatient intravenous (IV) antibiotics before improving over the course of five to seven days. The swelling and redness usually involve one or both hands or feet, and sometimes extends up the forearm, but there is no associated cyanosis or pruritus nor are her symptoms worsened by exposure to heat. She had been recently admitted to an outside hospital and treated with IV vancomycin and ceftriaxone over 2 days for presumed cellulitis. However, the patient reported no improvement and left the hospital against medical advice prior to presenting to our ED. At that point, the left hand was primarily affected with a “burning” sensation described as the “worst pain the patient had ever felt.”

There was no preceding fever and no recent trauma to her extremities. There was no joint or eye pain. The patient did not report hair loss or cold/heat intolerance. She had not been using any new soaps, detergents, creams or jewelry, and had no travel history. She does not take any medications or use recreational drugs. She reported no family history of rheumatologic disease.

On exam, the patient had normal vital signs, rated her pain as eight out of ten and appeared uncomfortable. There was an erythematous, warm, and exquisitely tender patch involving the palmar surface of the left hand and the first through fourth fingers along with a ten-centimeter linear lesion on the volar aspect of the left distal forearm. Flexion and extension of the left-sided fingers were limited due to pain. There were no secondary changes including scaling, crusting, fissures, ulcer or excoriation and no additional lesions involving the remainder of the extremities.

Laboratory evaluation showed a normal white blood cell count, hemoglobin, hematocrit and platelets. Mild elevations were noted in the erythrocyte sedimentation rate (ESR) (30 mm/hr, normal range 0–20 mm/hr) and C-reactive protein (CRP) (3.8 mg/dL, normal range 0–0.74 mg/dL). Creatinine kinase (CK) was normal and urine pregnancy negative.

## Significant findings

Episodic tender, warm, erythematous swelling of the extremity experienced by this patient is typical of erythromelalgia. Erythematous streaking on the volar surface of the left forearm (red arrow) and tender, warm, erythematous blanching swelling was present on the palmar hand (yellow arrow). Most patients with erythromelalgia also have lower extremity involvement including the dorsum or sole of the foot and toes.^1^

## Patient course

The rheumatology service was consulted in the ED to aid in diagnosis and pain management. Given a characteristic history and exam as well as reassuring limited laboratory evaluation, the patient was diagnosed with erythromelalgia. This patient, like the vast majority of patients, had intermittent symptoms of the extremities lasting minutes to days with a fluctuating distribution from unilateral or bilateral involvement.^1^–^4^ Her condition followed a chronic and progressive course and, as in many patients, symptoms can be constant and disabling.^1^

Our patient followed supportive therapy and use of naproxen as needed for pain, redness and/or swelling of extremities. She was referred to outpatient rheumatology and rehabilitation clinic, and on one-year follow up, has not suffered disease recurrence with little functional limitation.

## Discussion

Erythromelalgia, originally described in 1878, is a rare condition distinguished by recurrent episodes of intense burning pain, redness, swelling, and warmth localized to the distal extremities.^1^,^5^ It may present unilaterally or bilaterally – with the latter, a symmetric distribution is more common.^1^ Disease onset may occur spontaneously at any age and is about three times more common in females compared to males.^1^,^5^

The neuropathic component of erythromelalgia is due to dysregulated neuronal sodium channels in peripheral sympathetic and sensory nerves.^1^ The vascular component is attributed to maldistribution of small vessel blood flow and arteriovenous shunting which cause local hyperemia and tissue hypoxia.^3^,^4^ While there are no serologic or tissue markers for erythromelalgia, laboratory testing and/or punch biopsy may be useful in ruling out other conditions.^6^ There is also no clear association with autoimmune conditions, although erythromelalgia has been associated specifically with the development of myeloproliferative diseases.^1^ Early onset of erythromelalgia in childhood has been linked to an autosomal dominant mutation in the SCN9A gene encoding voltage-gated sodium channels of peripheral sensory and sympathetic neurons.^7^ Erythromelalgia is a chronic condition, and nearly one third of patients may experience progressive symptoms, (ie, more severe, frequent disease flares).^1^

Erythromelalgia is easily misdiagnosed as cellulitis, lymphangitis or Raynaud phenomenon.^1^,^2^,^5^ Emergency physicians should be aware that careful history is key in distinguishing the condition, since patients experience recurrent episodes of neuropathic pain following distinct triggers (ie, ambient heat, exercise).^1^ On exam, the physical distribution of erythromelagia involving the distal extremities and its recurrent, even progressive, episodes are characteristic features.^1^,^3^,^4^ Due to small vessel dysregulation, erythromelalgia in some cases may present as a violaceous lesion cool to the touch, similar to Raynaud phenomenon.^8^ Furthermore, attempted therapeutic cooling measures may trigger local vasospasm and acrocyanosis.^9^ Despite these similarities, there is a distinct pathologic process in each condition: whereas Raynaud phenomenon is due to disordered arteriolar vasoconstriction and triggered mainly by cold exposure, erythromelalgia is a manifestation of unregulated hyperemia that is primarily heat exposure-induced. 8

Erythromelalgia poses not only a diagnostic but therapeutic challenge with no known cure and often results in a significant impact on quality of life.^1^–^5^ If a presumptive diagnosis can be made in the ED setting, the first steps are to identify and avoid disease triggers such as excessive heat exposure. Nonpharmacologic (cooling) measures should be used in moderation, because prolonged fanning or icing can provoke painful acrocyanosis, maceration, and edema.^9^ Topical agents such as lidocaine patch and capsaicin, as well as non-steroidal anti-inflammatory drugs (NSAIDs) may be helpful.^10^,^11^ Patients should be referred to a dermatologist, rheumatologist, and/or pain management specialist for disease monitoring and initiation of systemic therapy, which may include aspirin and/or gabapentin.^12^,^13^ Lastly, rehabilitation programs may serve as an important therapeutic complement with focus on optimizing patients’ functional status and quality of life.^14^

This is a case illustration of a teenage patient with chronic erythromelalgia repeatedly misdiagnosed and treated for cellulitis. This is a rare but often progressive and disabling condition with underlying microvascular dysfunction (hyperemia and hypoxemia) and neuropathy. There are no definitive diagnostic tests and the condition may be mimicked by cellulitis, lymphangitis or Raynaud phenomenon, albeit with key differentiating features on history and exam described above. Management is supportive and targeted to optimizing patient quality of life, based on a combination of trigger avoidance, non-pharmacologic and pharmacologic therapy, and rehabilitation.

## Supplementary Information




