# Supplementary figures and images for: Not Another Presentation of Cellulitis: A Case Report of Erythromelalgia
Source: J Educ Teach Emerg Med. 2022 Jan 15;7(1):V31–4. doi: 10.21980/J8BD2K (PMC10358863; doi:10.21980/J8BD2K)

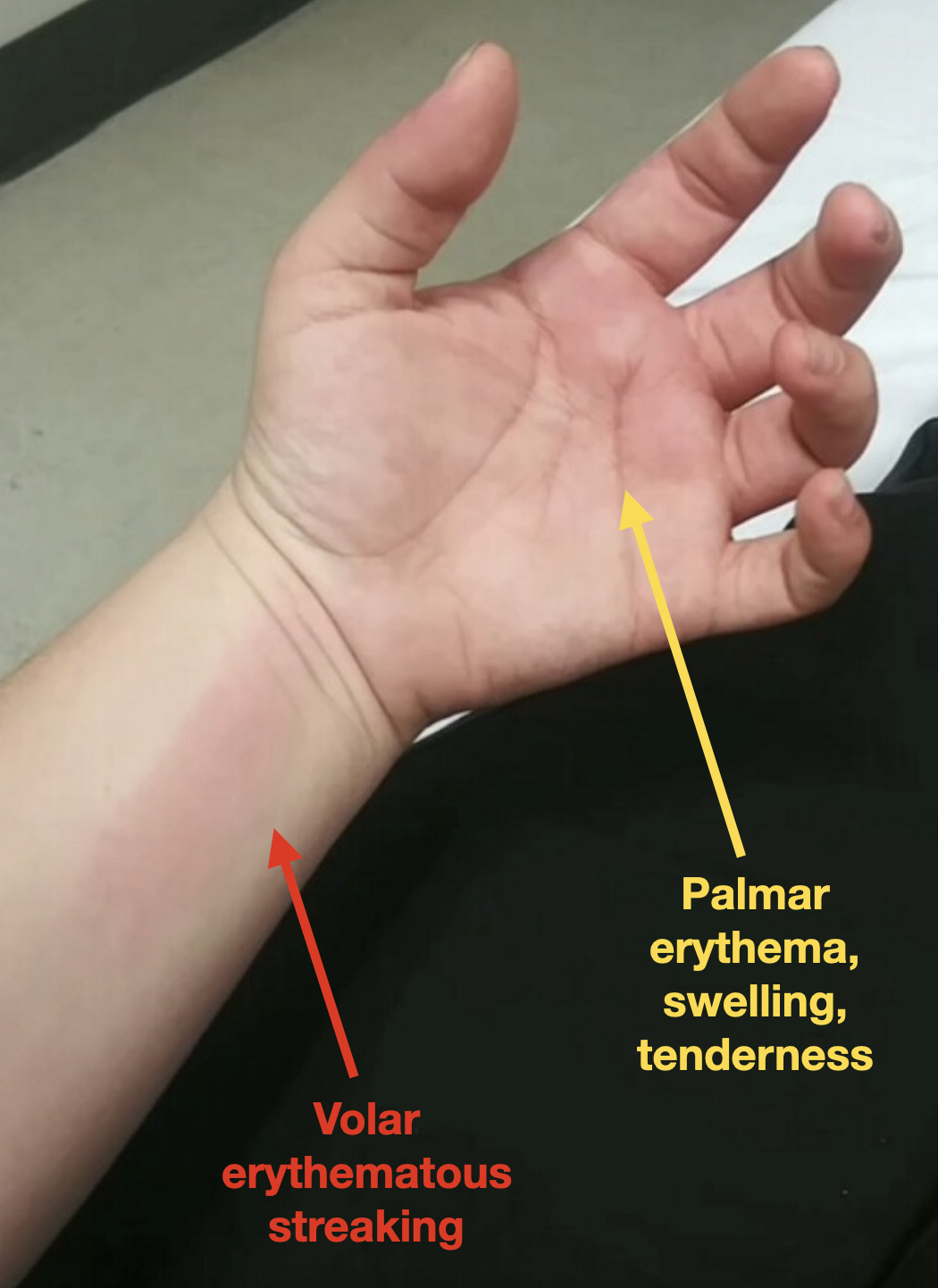

Supplement: Supplementary file 1 [file JETem-7-1-V31-supp1.jpg]

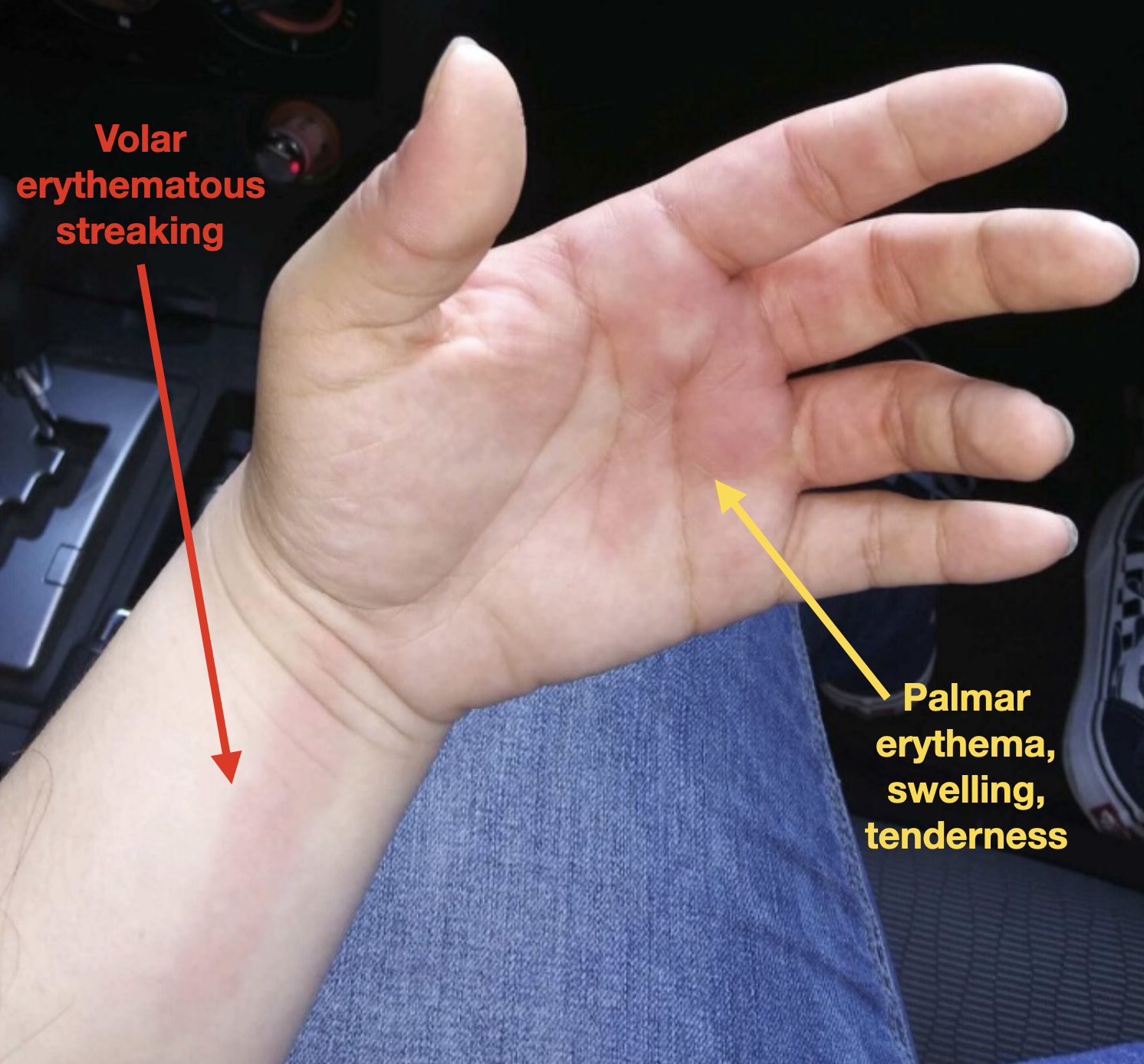

Supplement: Supplementary file 2 [file JETem-7-1-V31-supp2.jpg]
